# A Summary of Public Access Defibrillation Laws, United States, 2010

**Published:** 2012-03-15

**Authors:** Siobhan Gilchrist, Linda Schieb, Qaiser Mukhtar, Amy Valderrama, Paula Yoon, Comilla Sasson, Bryan McNally, Michael Schooley

**Affiliations:** Columbus Technologies and Services, Inc, Centers for Disease Control and Prevention; Centers for Disease Control and Prevention, Atlanta, Georgia; Centers for Disease Control and Prevention, Atlanta, Georgia; Centers for Disease Control and Prevention, Atlanta, Georgia; Centers for Disease Control and Prevention, Atlanta, Georgia; University of Colorado, Denver, Colorado; Emory University School of Medicine, Atlanta, Georgia; Centers for Disease Control and Prevention, Atlanta, Georgia

## Abstract

**Introduction:**

On average, less than 8% of people who experience an out-of-hospital cardiac arrest survive. However, death from sudden cardiac arrest is preventable if a bystander quickly retrieves and applies an automated external defibrillator (AED). Public access defibrillation (PAD) policies have been enacted to create programs that increase the public availability of these devices. The objective of this study was to describe each state's legal requirements for recommended PAD program elements.

**Methods:**

We reviewed state laws and described the extent to which 13 PAD program elements are mandated in each state.

**Results:**

No jurisdiction requires all 13 PAD program elements, 18% require at least 10 elements, and 31% require 3 or fewer elements. All jurisdictions provide some level of immunity to AED users, 60% require PAD maintenance, 59% require emergency medical service notification, 55% impose training requirements, and 41% require medical oversight. Few jurisdictions require a quality improvement process.

**Conclusion:**

PAD programs in many states are at risk of failure because critical elements such as maintenance, medical oversight, emergency medical service notification, and continuous quality improvement are not required. Policy makers should consider strengthening PAD policies by enacting laws that can reduce the time from collapse to shock, such as requiring the strategic placement of AEDs in high-risk locations or mandatory PAD registries that are coordinated with local EMS and dispatch centers. Further research is needed to identify the most effective PAD policies for increasing AED use by lay persons and improving survival rates.

## Introduction

Each year, emergency medical services (EMS) personnel respond to approximately 300,000 out-of-hospital cardiac arrests (OHCAs), yet, on average, less than 8% of people who experience an OHCA survive to hospital discharge (1). This rate has remained unchanged for more than 30 years (2). A person experiencing cardiac arrest is 2 to 3 times more likely to survive if a bystander applies an automated external defibrillator (AED) before EMS arrival (3-5). However, the rate of AED use before EMS arrival is only 2% for all OHCA events and 8% for OHCA events in a public setting (6).

A survey of state EMS agency directors indicated that a lack of legislation authorizing the use of AEDs by nonmedical first responders impeded state attempts to increase the accessibility of AEDs in public locations (7). The American Heart Association (AHA) has since promoted structured public access defibrillation (PAD) programs to allow nonmedical first responders and lay bystanders to use AEDs (8). The Cardiac Arrest Survival Act of 2000 required the establishment of federal guidelines for AED placement in federal facilities and provides immunity from civil suits to any person who uses an AED in an emergency (9). In 2006, the AHA recommended that states adopt a legislative approach to support community lay rescuer PAD programs (10). As a result, states have enacted laws to increase the availability and use of AEDs, to limit liability from AED use, and to require businesses, schools, and others to implement PAD programs (11).

The AHA and other organizations provide Internet-accessible comparisons of state PAD laws. To date, the extent to which state laws incorporate key PAD program elements has never been examined nationally. The objective of this study was to describe each state's legal requirements for recommended PAD program elements.

## Methods

We conducted a document review of laws in the 50 states and the District of Columbia (DC) (51 jurisdictions) in effect as of January 1, 2010. Search terms included *automated external defibrillator, automatic external defibrillator, AED, public access defibrillation, PAD, cardiopulmonary resuscitation, CPR, cardiac arrest, sudden cardiac arrest, public access, emergency medical service, emergency response, Good Samaritan, civil liability,* and *immunity*. We excluded laws regulating AEDs associated with EMS and other professional certification or medical facilities. From October 2010 through May 2011, we searched and reviewed legislation enacted in the 2010 legislative session, statutes, and relevant agency regulations, hereinafter referred to as laws. We used 2 legislative search engines, Westlaw (Thomson Reuters, Eagan, Minnesota) and State Net (LexisNexis, Sacramento, California), and cross-referenced our findings with the Internet legislative databases and administrative code Internet sites of each jurisdiction.

Using the 2006 AHA recommendations as a guide, we identified 6 categories of PAD program elements (a total of 13 elements) (10): 1) targeted AED site placement to maximize use; 2) ongoing basic life support cardiopulmonary resuscitation (CPR) and AED training of anticipated rescuers (eg, staff likely to be present during business hours) through an approved course; 3) maintenance and testing of the AED device to ensure continued functionality; 4) coordination with EMS and a medical provider, to include providing notification of or registering the type of AED and its location with the EMS communications or dispatch center in the EMS response area, by calling 9-1-1 when an AED is used, and through oversight by a medical professional with emergency response expertise; 5) continuous quality improvement through the use of a written medical emergency response plan or medically approved protocol, reporting each OHCA and clinical use of a PAD for evaluation by the program’s medical director, and use of the findings to improve program performance (3,11,12); and 6) limited liability for AED users and others. We collected the text and citations of each law and coded for 13 variables. We assigned a categorical value (Yes [Y], No [N], Conditional [C]) to the jurisdiction for each PAD program element according to the following: Y, if the PAD element is explicitly required for all PAD programs; N, if the PAD element is not required or provided for by law for all PAD programs; and C, for laws that encourage or require the adoption of the PAD element only in some locations, or only if certain conditions are met, or if it is solely a condition of civil immunity. To determine the extent to which a given jurisdiction has a comprehensive PAD policy, we calculated the total number of PAD elements with Y values out of a possible 13 elements. We conducted a descriptive analysis, summarizing the laws across 51 jurisdictions.

## Results

As of 2010, all jurisdictions have enacted laws addressing at least 1 PAD element, although no jurisdiction required all 13 elements (Table). The most common PAD elements regulated by law are civil immunity, training of anticipated rescuers, maintenance and testing, activation of 9-1-1, and registration of the AED. Continuous quality improvement is the least common PAD element regulated by law.

### Comprehensiveness of PAD legislation

For the 51 jurisdictions, the median number of required PAD elements was 6 (interquartile range, 3.0-8.5). Nine jurisdictions (18%) have the most comprehensive laws, requiring 10 or more elements for all PAD programs (Figure). In most jurisdictions, many PAD elements are required only for PAD programs in targeted locations, encouraged, conditional, or not provided for by law for all PAD programs. Sixteen jurisdictions (31%) require 3 or fewer PAD elements for all programs.

**Figure.**
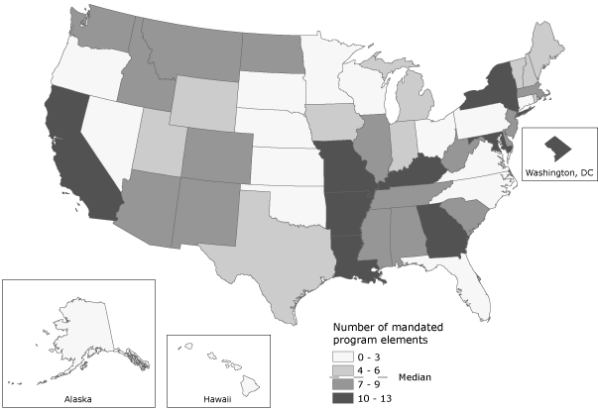
Summary of mandated public access defibrillation program elements in the United States as of January 1, 2010.

### Targeted AED site placement

Laws supporting targeted AED placement exist in 29 jurisdictions; 20 jurisdictions mandate placement of AEDs in specific locations, of which fitness facilities (11 jurisdictions) and schools or school-sponsored athletic events (9 jurisdictions) were the most common (Table). Five jurisdictions require AEDs in schools or law enforcement vehicles if funds are available, and 9 jurisdictions authorize or encourage AEDs in schools, public buildings, state parks, fitness facilities, or law enforcement vehicles. Few jurisdictions specify where the AED should be placed in a building, such as in a clearly visible or central location. Laws in 8 jurisdictions impose a civil penalty for noncompliance with the requirement to obtain an AED or in some cases for failure to comply with training, maintenance, or other requirements. The agency responsible for enforcement varies by jurisdiction. Only 15 jurisdictions that require AEDs in specific locations provide by law for the allocation of funds or other means of obtaining funding to purchase AEDs, although these laws do not typically cover maintenance costs.

### Training

In 28 (55%) jurisdictions (Table), anticipated rescuers at all AED sites must be trained; 8 of these jurisdictions also require PAD programs in targeted locations. Three states — Massachusetts, New Jersey, and New York — require any person who operates an AED to have completed a training course. Anticipated rescuer training is encouraged or is a condition of civil immunity in 15 (29%) jurisdictions. Nevada and Rhode Island require trained staff in fitness facilities, whereas for any other AED location having trained staff on site is a condition of immunity. Eight jurisdictions have no training requirement.

### Maintaining the AED device

In 31 (61%) jurisdictions, the law explicitly requires the person who acquires the AED or another entity to maintain and test the device according to the manufacturer's specifications (Table). Seven jurisdictions either encourage maintenance and readiness-for-use checks or require them as a condition of civil immunity. One state, Connecticut, requires only public golf courses to acquire and maintain AEDs. Twelve jurisdictions have no statutory provision for maintenance and testing. Tampering with or destroying an AED is a misdemeanor in Florida, Iowa, and Tennessee; this offense is limited to schools in Tennessee.

### EMS/medical coordination

In 30 (59%) jurisdictions, the entity that sells, supplies, or acquires the AED device must provide notification of or register the AED with the EMS system (Table). In 2 of these jurisdictions (DC and Maryland), PAD programs must also be certified by the agency that oversees the registration process. Three jurisdictions also require EMS notification when the AED is moved or removed. Five jurisdictions either encourage notification or require it as a condition of civil immunity. Laws in 29 (57%) jurisdictions require the person who uses an AED during a medical emergency to call 9-1-1 and activate the EMS system. In 4 states calling 9-1-1 is a condition of civil immunity for the AED user or the entity responsible for the AED placement. Fewer than half of all jurisdictions require or encourage medical provider oversight of the PAD programs. In 22 jurisdictions (43%), each clinical use of an AED must be reported; reporting clinical use is a condition of immunity in Colorado and Pennsylvania.

### Continuous quality improvement

Of 13 jurisdictions that require a PAD program facilitator to develop a medical emergency response plan or protocol for all AED sites, only 7 explicitly require written emergency response plans (Table). Two jurisdictions require emergency response plans or protocols only for select AED sites. Twenty-five percent of all jurisdictions require the PAD program facilitator (or other) to develop a quality improvement plan that includes an evaluation of each OHCA event.

### Immunity

In 41 (80%) jurisdictions, untrained lay rescuers are protected from civil action, typically provided that the lay rescuer acted in good faith as an ordinary, prudent person in similar circumstances. Seven jurisdictions protect lay rescuers from civil action provided they have met certain additional conditions, such as calling 9-1-1. Three jurisdictions (New Jersey, New York, and Rhode Island) provide civil immunity only to lay rescuers who have received CPR training. Twenty jurisdictions provide immunity to the person or entity that acquires the PAD. Twenty-four jurisdictions protect the AED acquirer from liability if additional PAD elements, such as maintaining the device, are met. Fewer than half of all jurisdictions provide limited immunity to the medical authority that oversees the program or to the owner, manager, or renter of the premises where the AED is installed.

## Discussion

This is the first comprehensive national analysis of state legal requirements of recommended PAD program elements. Our findings indicate that, in most jurisdictions, the critical elements necessary to sustain and ensure AED functionality are either missing from state laws or are not required of all PAD programs.

Less than 8% of people who have cardiac arrest will survive; however, this survival rate could be improved by increasing the use of AEDs in the community (2). A recent study in Japan found that increasing the number of available AEDs nationwide from 1 to 4 per square kilometer more than doubled the proportion of patients who were alive 1 month after the OHCA with minimal neurological impairment compared with those who did not receive a shock with an AED (13). To achieve optimal public health benefits of PAD programs, AEDs should be well marked, easy to access, and strategically placed in high-risk locations, such as transportation hubs, high-density public areas, sites with EMS response times longer than 5 minutes, and sites with an expected incidence of at least 1 cardiac arrest every 5 years (10,13-17). In addition, national organizations recommend the purchase and use of AEDs in emergency response planning in schools and fitness facilities (18,19). Despite these life-saving recommendations, few states have laws that require PAD programs in high-risk or high-density locations (Table).

An analysis of AED use rates in municipal buildings in Copenhagen found that PADs implemented through local or political initiatives were not used because of a low incidence of cardiac arrest at the site and a lack of accessibility to the AEDs by the general public (15). OHCA is not a reportable event in any US jurisdiction; few communities are able to plan an effective response because they lack the data to identify high-risk populations and locations or to evaluate if existing PAD programs are properly deployed (20). Ongoing evaluation of AED use rates in relation to where OHCAs actually occur is necessary to determine the optimal locations for PAD programs; however, we found that laws in 75% of the states do not mention quality improvement or evaluation of AED use. Policy makers should consider authorizing mandated reporting of OHCAs through standardized data collection systems such as the Cardiac Arrest Registry to Enhance Survival (CARES) program (21), the National EMS Information System (NEMSIS) (22), and the Resuscitation Outcomes Consortium (ROC) out-of-hospital cardiac arrest population-based registry (23). Such information can be used to track OHCA outcomes associated with AED use, to determine which EMS elements of the PAD programs are functioning properly, and to identify changes to PAD policies that can improve survival rates.

More than 200,000 AEDs are purchased annually for public use in the United States (24). However, our findings showed that more than 40% of jurisdictions do not require EMS notification or device registration of the AED location, suggesting that AEDs in many locations — including schools and other targeted sites — cannot be traced for recall. In 2005 alone, more than 50,000 AED devices were affected by advisories issued by the Food and Drug Administration (FDA). Between 1996 and 2005, 21.2% of AEDs were affected by recall advisories in 9 of the 10 years, and accessories, such as batteries, pads, and cables, were recalled in 7 of those years. In addition, 370 deaths were associated with an AED malfunction in the same time period (24). Coordinating the PAD program with an EMS agency is critical because a link with EMS or the 9-1-1 dispatch service can be used to inform AED owners about FDA-issued advisories and device recalls. Incorporating newer technologies such as global positioning system (GPS) and Next Generation 9-1-1 into existing and future AEDs should be considered to improve AED retrieval and maintenance.

A 2006 survey of PAD programs established in business, educational, and community buildings located throughout Johnson County, Iowa, found that after 2 years no site complied with all the AHA recommendations for community lay rescuer PAD programs (25). This study revealed multiple deficiencies in and barriers to adherence to the AHA guidelines, such as lack of access to and notification of the AED location, failure to replenish batteries, expired pads, scheduling of maintenance checks either infrequently or not at all, and limited to no funds for AED upkeep. At the time this study was conducted, Iowa law did not require AED maintenance. In 2010, we found that only 60% of all jurisdictions require AED maintenance, less than half require medical oversight of the program, and only 1 in 4 jurisdictions requires continuous quality improvement planning, indicating that PAD programs in many communities are at risk of failure. Further research evaluating facilitators and barriers to adherence to PAD program elements comparing jurisdictions with comprehensive PAD legislation to less regulated jurisdictions should help to identify the most effective policies for sustaining PAD programs.

AHA recommends that states provide immunity from civil liability for lay rescuers who act "in good faith, without specific compensation, as a reasonable and prudent person with the same level of training would respond" in an emergency, regardless of whether the lay rescuer was trained to provide CPR or use an AED (10). Good Samaritan laws provide this immunity by restricting the circumstances under which a lay rescuer can be sued for civil damages, thereby facilitating the use of AEDs by lay bystanders witnessing a cardiac arrest. Similarly, laws that protect PAD program facilitators from liability make it easier for businesses, schools, organizations, and others to implement PAD programs. We identified 3 states that do not provide immunity to untrained lay rescuers; such policies could impede efforts to use AEDs even though evidence shows that untrained lay persons can apply an AED safely and effectively. We also found that many jurisdictions have policies with conditional or no liability protection for AED acquirers and PAD medical directors, which may affect an organization's decision to purchase an AED or implement a PAD program. Concerns about the liability risks of implementing a PAD program were raised in a survey of Florida fitness club owners and managers, leading the study authors to conclude that a carefully designed, implemented, and operated PAD program may be the best risk management strategy (26). However, assessments of the legal risks associated with AEDs have found litigation arising primarily from not having a readily available AED and trained staff on the premises when a cardiac arrest occurs (11,27-29). Jurisdictions that confer broader liability protection on PAD program facilitators are more likely to have the flexibility to implement PAD programs in sites with a high risk of OHCA rather than placing them in low-risk areas in reaction to concerns about litigation.

The descriptive nature of this analysis limits our ability to discern whether comprehensive PAD policies are effective in saving lives. We were unable to associate cardiac arrest survival rates with the strength of a state policy or to assess the extent to which PAD programs are properly implemented in the states. Therefore, we are unable to assess to what extent PAD policies underlie geographic differences in cardiac arrest survival rates. Studies in the United States and Canada have shown a range of OHCA survival rates, from 7% to approximately 40% in some areas (1,3,20). Future studies assessing the number and locations of AEDs and PAD programs per state, as well as analyzing AED use and OHCA outcomes, are needed to assess whether comprehensive PAD policies are associated with improved survival rates. Our analysis was limited to state laws; therefore, we do not know the extent to which municipal ordinances requiring more PAD elements than required by state law improve PAD effectiveness. Finally, our review did not capture the actual rate of enforcement of PAD policies or the use of economic incentives to purchase AEDs.

Although all states and the District of Columbia have enacted laws to make PAD programs more widespread, policies in many jurisdictions leave these programs at risk of failure because critical elements necessary to ensure AED functionality are not always required. Policy makers should consider strengthening PAD policies by enacting laws that can reduce the time from collapse to shock, such as requiring the strategic placement of AEDs in high-risk locations or mandatory PAD registries that are coordinated with local EMS and dispatch centers. Further research is also needed to identify the most effective PAD policies for increasing AED use by lay persons and improving survival rates.

## Figures and Tables

**Table.** Summary of State and District of Columbia Public Access Defibrillation Statutes and Regulations in Effect in 51 US Jurisdictions, January 2010

PAD Element	**No. of Jurisdictions **		

	PAD Element Explicitly Required by Law	PAD Element Encouraged or Required by Law Under Certain Conditions	PAD Element Not Required or Provided For by Law
**Placement**			
PAD programs in specified locations (eg, fitness facilities)	20	9	22
**Training**			
Anticipated rescuers must be trained in CPR and AED use	28	15	8
**Maintenance and testing**			
Person who acquires an AED (or other) must maintain and test the AED	31	8	12
**EMS/medical coordination**			
Notification or registration of the AED with state or local EMS (or equivalent)	30	5	16
Activate 9-1-1 EMS	29	4	18
Report the clinical use of an AED to EMS (or equivalent)	22	2	27
Oversight by a licensed physician or medical authority	21	3	27
**Continuous quality improvement and planning**			
Written emergency response plans or medically approved protocols	13	2	36
Plan to evaluate all OHCA events	13	0	38
**Good Samaritan civil immunity**			
For untrained and trained lay rescuers	41	7	3
For AED acquirers^a^	20	24	7
For program directors^b^	21	11	19
For owner, manager, or renter of the premises where the AED is installed	14	6	31

Abbreviations: PAD, public access defibrillator; CPR, cardiopulmonary resuscitation; AED, automated external defibrillator; EMS, emergency medical services; OHCA, out-of-hospital cardiac arrest.

a AED acquirers include the person or entity that provides the AED site placement.

b Program directors include licensed physicians and medical authorities.
